# The impact of RNA extraction method on accurate RNA sequencing from formalin-fixed paraffin-embedded tissues

**DOI:** 10.1186/s12885-019-6363-0

**Published:** 2019-12-05

**Authors:** Michal Marczyk, Chunxiao Fu, Rosanna Lau, Lili Du, Alexander J. Trevarton, Bruno V. Sinn, Rebekah E. Gould, Lajos Pusztai, Christos Hatzis, W. Fraser Symmans

**Affiliations:** 1 Yale Cancer Center, Yale School of Medicine, New Haven, CT USA; 2 Data Mining Division, Silesian University of Technology, Gliwice, Poland; 3 Department of Pathology and Translational Molecular Pathology, The University of Texas MD Anderson Cancer Center, Houston, TX USA; 4 Institute of Pathology, Charité Universitätsmedizin Berlin, Berlin, Germany

**Keywords:** RNA extraction, RNA sequencing, FFPE-based clinical assay

## Abstract

**Background:**

Utilization of RNA sequencing methods to measure gene expression from archival formalin-fixed paraffin-embedded (FFPE) tumor samples in translational research and clinical trials requires reliable interpretation of the impact of pre-analytical variables on the data obtained, particularly the methods used to preserve samples and to purify RNA.

**Methods:**

Matched tissue samples from 12 breast cancers were fresh frozen (FF) and preserved in RNA*later* or fixed in formalin and processed as FFPE tissue. Total RNA was extracted and purified from FF samples using the Qiagen RNeasy kit, and in duplicate from FFPE tissue sections using three different kits (Norgen, Qiagen and Roche). All RNA samples underwent whole transcriptome RNA sequencing (wtRNAseq) and targeted RNA sequencing for 31 transcripts included in a signature of sensitivity to endocrine therapy. We assessed the effect of RNA extraction kit on the reliability of gene expression levels using linear mixed-effects model analysis, concordance correlation coefficient (CCC) and differential analysis. All protein-coding genes in the wtRNAseq and three gene expression signatures for breast cancer were assessed for concordance.

**Results:**

Despite variable quality of the RNA extracted from FFPE samples by different kits, all had similar concordance of overall gene expression from wtRNAseq between matched FF and FFPE samples (median CCC 0.63–0.66) and between technical replicates (median expression difference 0.13–0.22). More than half of genes were differentially expressed between FF and FFPE, but with low fold change (median |LFC| 0.31–0.34). Two out of three breast cancer signatures studied were highly robust in all samples using any kit, whereas the third signature was similarly discordant irrespective of the kit used. The targeted RNAseq assay was concordant between FFPE and FF samples using any of the kits (CCC 0.91–0.96).

**Conclusions:**

The selection of kit to purify RNA from FFPE did not influence the overall quality of results from wtRNAseq, thus variable reproducibility of gene signatures probably relates to the reliability of individual gene selected and possibly to the algorithm. Targeted RNAseq showed promising performance for clinical deployment of quantitative assays in breast cancer from FFPE samples, although numerical scores were not identical to those from wtRNAseq and would require calibration.

## Background

Most gene expression signatures of breast cancer currently employ RT-PCR amplification or direct hybridization to oligonucleotide probes [1]. RNA sequencing (RNAseq) is a rapidly emergent technology for translational research and potential clinical use [2], supported by strong cross-platform concordance with existing technologies such as microarrays. For example, expression from whole transcriptome RNAseq (wtRNAseq) and microarrays prepared from 57 fresh frozen (FF) breast cancers demonstrated strong correlation (*r* > 0.9) for many genes, including *ESR1* (estrogen receptor), *PGR* (progesterone receptor) and *ERBB2* (HER2 receptor), and established multigene signatures such as EndoPredict and OncotypeDX (*r* > 0.95) [3]. Based on such promising analytical performance, attention should be given to development of evidence-based standard operating procedures for clinical-level implementation with routine formalin-fixed paraffin-embedded (FFPE) tumor samples, for both targeted and wtRNAseq applications.

Several pre-analytical methods have been proposed to overcome challenges with low quality or low quantity RNA derived from FFPE specimens [4]. Overall, gene expression levels from RNAseq of FFPE and matched FF tumor samples are strongly correlated, irrespective of storage time and tissue type [5–7]. However, some genes are more variable (≥ 2-fold expression difference between FFPE and FF samples), largely independent of the tissue type [8]. In addition, extended delay prior to fixation can impact the measurements of individual gene expression levels [9]. Protocols that enrich for messenger RNA transcripts (mRNA) by depleting the predominant ribosomal RNA (rRNA) perform well with FFPE samples [10], and targeting the 3′ end of mRNA can achieve similar results [11]. In a recent study, we evaluated which wtRNAseq library preparation protocols provide the best calibration between FFPE and FF samples. We identified the RNase H-based KAPA kit for rRNA depletion and sequencing library preparation as our preferred FFPE library preparation protocol for subsequent projects [12].

**Fig. 1:**
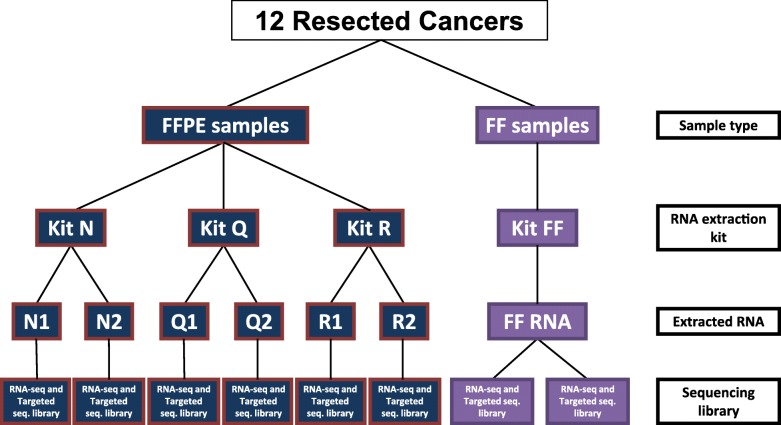
Design of the study

It is equally important to credential RNA extraction since this is potentially an important pre-analytical factor, with several methods offered in commercially available kits. In this study, we evaluated three commercial kits for FFPE biopsy samples (Fig. 1), each representing a different method for RNA extraction, by comparing the RNA quality and concordance of gene expression measurements from FFPE with the matched FF samples as gold standard. Replicate experiments allowed independent estimation of the various contributions to the analytical noise of the assay. This study design was applied to wtRNAseq assay and to a targeted RNAseq assay that quantifies transcript target expression at considerably higher read depth [13].

## Methods

### Tissue samples

A specialized breast pathologist (MD Anderson Cancer Center) collected research tissue samples from freshly resected invasive breast cancers at the time of intra-operative specimen evaluation (IRB protocol LAB08–0824) from 12 treatment-naïve, stage I-III breast cancers that were selected to represent the main biological subtypes (Table 1). We used a procedure to negate effects from intratumoral heterogeneity: dicing, mixing and evenly dividing the tissue fragments into two conditions of preservation [14]. Half of each sample was placed into RNA*later* (Qiagen) at room temperature, then held in a 4 °C refrigerator (6–72 h) and after that stored frozen at − 80 °C until use (FF). The other half was placed into 10% neutral buffered formalin solution, fixed at room temperature (8–72 h) and then processed routinely into a paraffin embedded tissue block (FFPE). All samples were stored until we had compiled the cohort and were ready to begin the study (21–330 days). Then, the FFPE blocks were sectioned to prepare an H&E stained slide and unstained sections (5 μm thick) on glass slides for RNA extraction.

**Table 1:** Clinical-pathologic characteristics of the 12 breast cancer samples in this study

Patient	Age	Grade	Stage	ER status	PR status	HER2 status
16H	[50–60)	3	III	P	P	N
16 J	[40–50)	1	II	P	P	N
16 K	[40–50)	2	II	P	P	N
16 W	[70–80)	2	I	P	N	N
17E	[60–70)	2	I	P	P	N
18Y	[40–50)	2	I	P	P	P
19A	[50–60)	2	II	P	P	N
19D	[80–90)	3	III	P	P	N
19G	[50–60)	2	II	P	N	N
19 K	[50–60)	2	II	P	P	N
19 M	[40–50)	3	I	P	P	N
19O	[50–60)	2	II	P	P	N

### RNA extraction protocols

The FF sample was thawed and RNA was extracted using the Qiagen RNeasy kit [12, 14]. For FFPE samples, RNA was extracted from adjacent tissue sections for each of three commonly-used commercial kits: N – Norgen (FFPE RNA purification Kit, Norgen, Thorold, Canada), Q – Qiagen (AllPrep DNA/RNA FFPE kit, Qiagen, Valencia, CA) and R – Roche (High Pure FFPE RNA Micro Kit, Roche, Indianapolis, IN). Two replicate RNA extractions were obtained per sample for each kit.

DNase I treatment was applied during both the FF and FFPE RNA isolation protocols. RNA concentration was quantified by Nanodrop (Nanodrop Technologies, Wilmington, DE). The RNA quality was analyzed using the Agilent 2100 Bioanalyzer (Agilent Technologies, Palo Alto, CA) to produce an electrophoresis trace from which the RNA integrity number (RIN) and DV200 index were calculated using the 2100 Expert Software (Agilent Technologies). RIN is an algorithm used to estimate the integrity of RNA based on a combination of different features. RIN varies from 1 to 10, where 10 means perfect RNA integrity [15]. DV200 metric is the percentage of RNA fragments longer than 200 nucleotides and was found as a reliable determinant for RNA quality [16].

### Whole-transcriptome and targeted RNA sequencing

Whole transcriptome RNAseq libraries were prepared from all samples using RNA HyperPrep kit with RiboErase (HMR) (Kapa Biosystems, Wilmington, MA), as we previously described [12]. Sequencing was performed using Illumina HiSeq 4000 (Illumina, San Diego, CA), with 6 libraries pooled per lane including FF and FFPE samples. Fragment protocols differed, 94 °C for 5 min for FF and 85 °C for 6 min for FFPE, in order to balance the number of sequencing reads per library. Targeted RNAseq sequencing libraries were prepared using a customized micro-droplet based protocol as described previously [13]. Droplet-generation was performed using RainDance Source system (BioRad, Hercules, CA) and was followed by a one-step RT-PCR reaction (1st PCR) to target the regions of interest with our custom multiplex primer set. A 2nd PCR step incorporated RainDance DirectSeq primers for sample indexing and Illumina specific adapters for cluster generation/sequencing. The resultant libraries were then quantified by Bioanalyzer, and sequenced by Illumina MiSeq (Illumina, San Diego, CA), with up to 40 libraries pooled per flow cell.

### Pre-processing of sequencing reads, alignment and quantification

Raw reads were assessed for quality using FastqQC v0.11.5 [17] and adapter sequences were identified and removed using Trimmomatic v0.36 [18]. Remaining reads were aligned against the human genome (hg38) using STAR v2.5.3a [19] with two-pass mode and default parameters. The alignment quality measures and coverage along transcripts was assessed using RSeQC v2.6.4 [20]. Transcript integrity score (TIN) captures the uniformity of sequence coverage for each transcript, and median TIN provides a measurement of RNA integrity [21]. TIN varies from 0 to 100, where 100 means perfect RNA integrity. Distance along transcript was normalized to a 0–100% range and summarized across transcripts for each sample. Transcripts were assigned into one of 4 groups based on their length distribution (length of all exons within given transcript). Gene expression was quantified using RSEM v1.3.0 [22] with option for strand-specific RNA library. Only reads in exonic regions were used to calculate gene expression levels. ENSEMBL release 91 was used to annotate reads within human genes. Finally, expression levels were normalized using a panel of 10 reference genes used in SET_ER/PR_ signature [13] and log-transformed. Only protein coding genes were selected for statistical analysis, and genes not expressed in all samples within the same RNA extraction kit were removed, resulting in 18,695 genes in the final analysis.

### Selected molecular signatures in breast cancer

Three mRNA-based gene signatures were selected to compare RNA extraction kits. EndoPredict measures 8 genes (AZGP1, BIRC5, DHCR7, IL6ST, MGP, RBBP8, STC2, UBE2C) relative to 3 reference genes (CALM2, OAZ1, RPL37A), and is performed as a commercial test on a RT-PCR platform [23]. The recurrence score (RS; OncotypeDx commercial assay) measures 16 informative genes (AURKA, BAG1, BCL2, BIRC5, CCNB1, CD68, CTSV, ERBB2, ESR1, GRB7, GSTM1, MKI67, MMP11, MYBL2, PGR, SCUBE2) relative to 5 normalizers (ACTB, GAPDH, GUSB, RPLP0, TFRC) [24]. The EndoPredict and RS scores were calculated using the *genefu* package in R [25]. The SET_ER/PR_ index (for sensitivity to endocrine therapy) was developed from Affymetrix microarrays to measure transcriptional activity related to estrogen and progesterone receptors in breast cancer [26]. It uses 18 informative genes (ABAT, ADCY1, AZGP1, CA12, CD2, CD3D, DNAJC12, ESR1, KCNE4, MAPT, MRPS30, NAT1, NPY1R, PDZK1, QDPR, SCUBE2, SLC39A6, STC2) relative to 10 reference genes (AK2, APPBP2, ATP5J2, DARS, LDHA, TRIM2, UBE2Z, UGP2, VDAC2, WIPF2) [13]. The SET_ER/PR_ index was calculated from log-transformed read counts from both whole transcriptome and targeted sequencing assays [13].

### Statistical analysis

We used principal component analysis (PCA) with Euclidean distance to evaluate the overall expression of protein-coding genes. Pearson correlation coefficient (r) was used to compare gene expression levels and molecular signature scores between samples. Spearman correlation coefficient (r_S_) was used to compare results of analysis between RNA extraction kits. Agreement between FF and FFPE samples was assessed using Lin’s concordance correlation coefficient (CCC) [27] using average measurements from technical replicates from each kit. Lin’s coefficient modifies the Pearson correlation coefficient by assessing not only how close scattered data are to the line of best fit (Correlation term ranging from − 1 to 1; higher is better) but also how far that line is from perfect agreement (Bias term ranging from 0 to 1; higher is better).

We compared RIN, DV200 and TIN indices of RNA quality between samples using linear modeling of paired data implemented in the *limma* R package [28]. Measurements from technical replicates were averaged prior to analyses. For each of two indices separately, the following model with two fixed effects was fitted:

$$ \mathit{\mathsf{Y}}=\mathit{\mathsf{Cancer}}+\mathit{\mathsf{Kit}} $$

where *Y* is a RIN, DV200 or TIN index, *Cancer* indicates tumor sample and *Kit* is the FFPE RNA extraction kit used or FF sample (reference). The *Kit* fixed effect term models difference in RNA quality between FFPE RNA extraction kits and matched FF sample. *P*-values obtained from linear model analysis were corrected for multiple testing using the Benjamini-Hochberg false discovery rate method.

Our study design allowed using linear mixed-effects (LME) model analysis to estimate the effects of sample type and RNA extraction kit on the reliability of the individual gene expression or molecular signature score. The model was implemented in *lme4* R package [29] with restricted maximum likelihood estimation. For each individual gene and molecular signature score, the following model with one fixed and two random effects was fitted:

$$ \mathit{\mathsf{Y}}=\mathit{\mathsf{Kit}}+\left(\mathit{\mathsf{Kit}}\ |\ \mathit{\mathsf{Cancer}}\right)+\left(\mathsf{1}\ |\ \mathit{\mathsf{RepWcancer}}\right) $$

where *Y* is a normalized log2 expression of individual gene or molecular signature score, *Kit* is the FFPE RNA extraction kit used or FF sample (reference), *Cancer* indicates tumor sample and *RepWcancer* groups replicates of the same tumor sample and RNA extraction kit. The fixed effect term of the model *Kit* estimates biases in expression level between FFPE RNA extraction kits and FF sample. The random intercept (*Kit* | *Cancer*) represents the variance in the FFPE Kit vs FF effect across cancer samples, while the term (1 | *RepWcancer*) represents the noise between replicates within each sample.

Individual gene expression was compared between FF and FFPE samples using *DESeq2* R package [30] for differential analysis. Prior to the analysis the measurements from technical replicates were averaged. For gene expression matrix the following model with two fixed effects was fitted:

$$ \mathit{\mathsf{Expression}}=\mathit{\mathsf{Cancer}}+\mathit{\mathsf{Kit}} $$

where *Expression* is a raw gene counts matrix, *Cancer* indicates tumor sample and *Kit* is the FFPE RNA extraction kit used or FF sample (reference). The *Kit* fixed effect term models difference in expression between RNA extraction kits. Differentially expressed genes (DEGs) were defined as Benjamini-Hochberg method adjusted *p*-value < 0.05.

For all other comparisons between FF and FFPE samples, e.g. RNA quality metrics, sequencing metrics or CCC values, nonparametric Mann-Whitney U-test was used. In all tests the significance level was set to 0.05.

## Results

### RNA quality

We compared three indices of RNA quality, RIN, DV200 and TIN, between FF and FFPE RNA extraction kits for 12 cancer samples (Additional file 1: Table S1). On average, RIN and DV200 show that the quality of RNA extracted from FFPE tissues was worse than from FF tissues (RIN: median for FF = 7.2, median for FFPE = 2.5; DV200: median for FF = 88, median for FFPE = 77; Additional file 2: Table S2). The three FFPE RNA kits were highly similar to each other, yielding low RIN (Kit N: median = 2.4, range = 2–7.1; Kit Q: median = 2.5, range = 1.9–4.6; Kit R: median = 2.5, range = 1.9–7) and DV200 (Kit N: median = 79.5, range = 57–90; Kit Q: median = 73, range = 63–87; Kit R: median = 83, range = 70–92) measures. DV200 of RNA from kit R was not significantly different than FF RNA. When comparing FFPE RNA extraction kits, kit N yielded higher quality RNA than kit Q, but not statistically significantly so. The DV200 of RNA from kit R was higher than from kits N and Q (5 and 7%, respectively; Additional file 2: Table S2).

On the other hand, TIN score that is calculated on genome aligned read files for each individual transcript, shows that the integrity of RNA extracted from FF tissues was worse than from FFPE (Additional file 3: Figure S1). Median TIN score was higher for FFPE samples than for FF (median for FF = 75.84, median for FFPE = 81.66) and the difference was statistically significant for all kits (Additional file 2: Table S2). Again, the three FFPE RNA kits were highly like each other, showing no statistically significant differences in median TIN (Kit N: median = 82.02, range = 79–83; Kit Q: median = 81.41, range = 76–84; Kit R: median = 81.27, range = 76–83).

### Quality of RNA sequencing reads

**Fig. 2:**
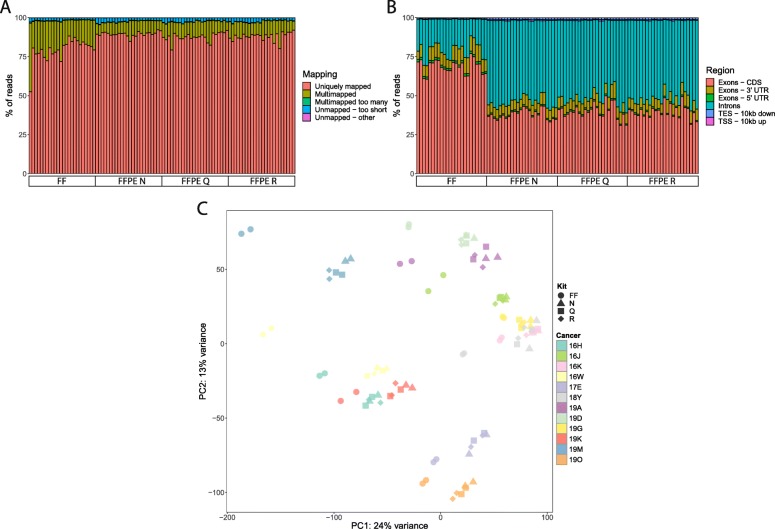
Mapping of reads to genome and gene expression quantification results for wtRNAseq data. **a** Mapping summary statistics from STAR aligner. **b** Distribution of genomic regions in which sequencing reads were aligned. **c** PCA analysis based on expression levels of all protein-coding genes

Sequence libraries from FFPE and FF samples were of similar quality (Additional file 4: Table S3), as we previously reported [12]. Specifically, the size ranged from 40 M to 100 M reads, were similarly distributed, and with high base quality (Q > 35) at all positions. The libraries from FF samples had higher levels of read duplication (Fold change(FC) = 1.65; *p* < 0.001), higher percentage of GC content (FC = 1.15; *p* < 0.001), and higher prevalence of Illumina adapter sequences (FC = 7.29; *p* < 0.001). After read alignment to the reference genome, FF samples had ~ 10% fewer uniquely mapped reads (Fig. 2a), higher proportion of multi-mapped reads, higher expression of protein-coding genes (FC = 1.69; *p* < 0.01), and more reads mapped to chromosomes 14 and 21. Interestingly, FFPE samples had more reads mapping to intronic regions of the genome (Fig. 2b). The normalized coverage along transcript was similar for all samples (Additional file 5: Figure S2A), except for a single library (FF sample 16 J). We observed a greater percentage of reads for miscellaneous RNAs and smaller percentage of reads for long non-coding RNAs for FF samples than FFPE (Additional file 5: Figure S2B). After normalization, gene expression measurements were comparable between all samples. PCA analysis based on 18,695 protein-coding genes shows the three FFPE kits cluster together, separately from FF samples, but within each cancer sample (Fig. 2c). However, the first two PCs that we plotted explain only 37% of variance, so we assume that there is an extra heterogeneity in the data not explained by sample type or cancer.

### FFPE extraction kits produced RNAseq results concordant with FF samples

**Fig. 3:**
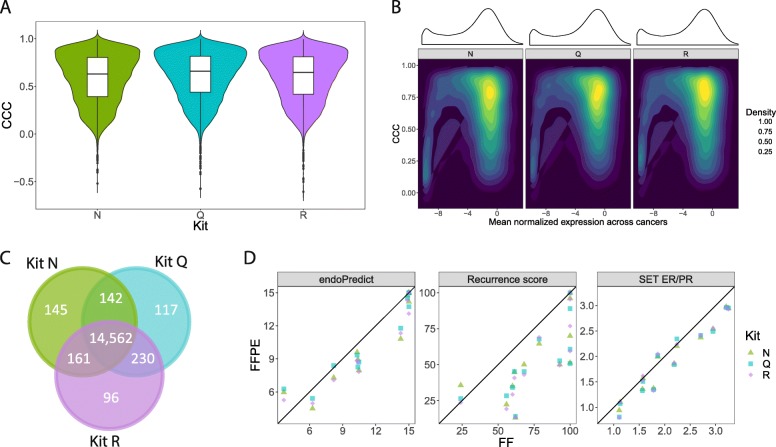
Concordance of gene expression between FFPE and FF samples for wtRNAseq data. **a** Distribution of concordance correlation coefficient (CCC) for all genes within each RNA extraction kit used. **b** Association between gene expression and CCC value. **c** High expression (normalized expression higher than − 7.5) and high concordant (CCC > 0.5) genes between different kits. **d** Concordance of molecular signatures scores for 3 FFPE kits in comparison to FF

The distributions of concordance correlation coefficient (CCC) in expression levels between FFPE and FF samples across all genes were comparable for each kit, without obvious bias (Fig. 3a, Table 2). Similarly, the CCC values between FFPE kits were highly correlated (r_s_ > 0.93 in all pairwise comparisons). Genes expressed at low levels generally had lower CCC (Fig. 3b). We compared the overlap between the three FFPE kits for genes with high expression level (normalized expression> − 7.5) and high concordance with FF (CCC > 0.5), and found that 94.2% genes were present in wtRNAseq data from all three FFPE kits (Fig. 3c) but only 25.9% for low expression and low concordance genes. With all FFPE kits, highly expressed genes exhibited higher CCC (Additional file 6: Figure S3A; CCC increase ~ 0.15; *p* < 0.001). The distribution of CCC per chromosome is similar except for chromosome Y (Additional file 7: Figure S4A). There were no regions in the genome with consistently lower CCC of gene expression between FFPE and FF samples using any of the three kits for FFPE samples (Additional file 7: Figure S4B).

**Table 2:** Descriptive statistics of concordance and LME analysis for all genes quantified by wtRNAseq in FFPE versus FF samples. Median values with median absolute deviation in brackets

Kit	Concordance analysis			LME model		Replicates
	CCC	R	Bias	Bias	Variance	Difference
N	0.629 (0.291)	0.856 (0.122)	0.778 (0.239)	0.269 (0.605)	0.123 (0.132)	0.133 (0.097)
Q	0.659 (0.266)	0.859 (0.116)	0.810 (0.206)	0.229 (0.561)	0.118 (0.129)	0.177 (0.106)
R	0.646 (0.278)	0.863 (0.113)	0.793 (0.226)	0.192 (0.619)	0.116 (0.127)	0.216 (0.124)

### Differences in gene expression measurements between FF and FFPE kits

More than half of genes were differentially expressed between FF and FFPE for all kits (Table 3; Additional file 8: Figure S5A). When we selected genes with log2-fold change (LFC) lower than − 1 or higher than 1 (doubling of expression), only around 1000 genes were significantly changed. The highest no. of DEGs was found for kit N, while for kit Q the smallest. The ratio of up- to down-regulated genes was close to 1, but when we selected genes with higher |LFC| there was much more genes with higher expression in FFPE than FF. Most of DEGs found (78.53%) are the same between kits (Additional file 8: Figure S5B). Again, the kit Q had the smallest number of unique DEGs. When comparing expression level between FFPE kits, there was only one differentially expressed gene between kits N and Q (Additional file 8: Figure S5C). Much more genes were differentially expressed between kits N and R, and Q and R (Additional file 8: Figure S5C).

**Table 3:** No. of differentially expressed genes (DEGs) in wtRNAseq

Contrast	|log2FC| > 0	|log2FC| > 0.5	|log2FC| > 1	|log2FC| > 2
FF vs N	12,387	6083	1125	31
FF vs Q	12,061	5361	757	14
FF vs R	12,383	6081	958	26
N vs Q	1	0	0	0
N vs R	774	20	0	0
Q vs R	212	0	0	0

### Gene expression signatures from RNAseq data

The scores for three selected breast cancer signatures calculated from wtRNAseq data were variably concordant between FF and FFPE samples (Fig. 3d). EndoPredict and SET_ER/PR_ were highly concordant (CCC > 0.9) without bias (Additional file 9: Table S4). However, the 21-gene Recurrence Score (CCC 0.49–0.56) had a bias for higher scores in FF samples, with score > 50 in 11/12 FF samples (Fig. 3d). The three kits for RNA extraction produced similar results for all signatures (Fig. 3d).

The individual genes within each of the molecular signatures were highly concordant between FF and FFPE with all three kits, when compared to all other genes (Additional file 10: Figure S6A). Informative genes were generally more concordant than reference genes, and this was similar with all RNA extraction kits (Additional file 10: Figure S6B). The three molecular signatures were each compared to 10,000 random signatures generated by averaging expression of the same number of randomly selected genes (within the same expression range). EndoPredict and SET_ER/PR_ had higher CCC than 90% of random signatures, whereas the Recurrence Score was below the median for random signatures, irrespective of RNA extraction kit (Additional file 10: Figure S6C).

### Technical variation from sample type and RNA extraction kit

**Fig. 4:**
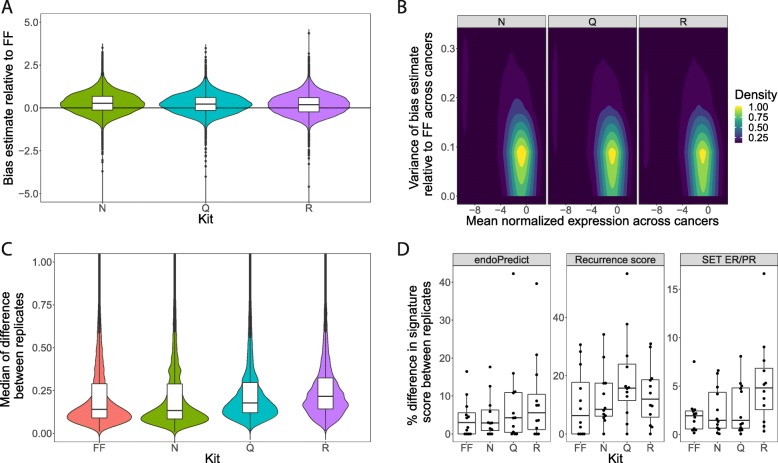
Technical variance and reliability of mRNA transcripts for wtRNAseq data. **a** Bias estimate component of LME model (closer to 0; better). **b** Variance component of LME model (smaller is better) vs gene expression level. **c** Distribution of median of difference in expression between replicates for all genes within each RNA extraction kit. **d** Percentage difference in molecular signature scores between technical replicates

A linear mixed-effects (LME) model, including expression data from technical replicates of each sample and RNA extraction condition, was fitted for each individual gene and molecular signature. The fixed effects of the model estimated the systematic bias between FFPE and FF samples, and the random effect estimated the variance of bias estimate compared to FF across cancers. All kits produced a small positive bias in expression between FFPE and FF samples (Fig. 4a, Table 2). Genes expressed at low levels had higher variance of bias across cancers (Fig. 4b). The bias for kit R was slightly less variable across cancers (Table 2), but kit N had the least variance between replicate FFPE samples, equivalent to FF samples (Fig. 4c). The same LME was fitted separately to 3 molecular signatures and showed negligible effect from RNA extraction kit (Additional file 9: Table S4). It appeared that Kit N was slightly less variable in technical replicates, and kit R slightly more (Fig. 4d), but differences were not statistically significant. The bias estimate of highly expressed genes was lower than for low expressed genes for all kits (Additional file 6: Figure S3B; Bias decrease ~ 0.45; *p* < 0.001) and the variance of bias estimate was also lower (Additional file 6: Figure S3C; Variance decrease ~ 0.2; *p* < 0.001).

### Whole transcriptome versus targeted RNAseq for SET_ER/PR_ index

**Fig. 5:**
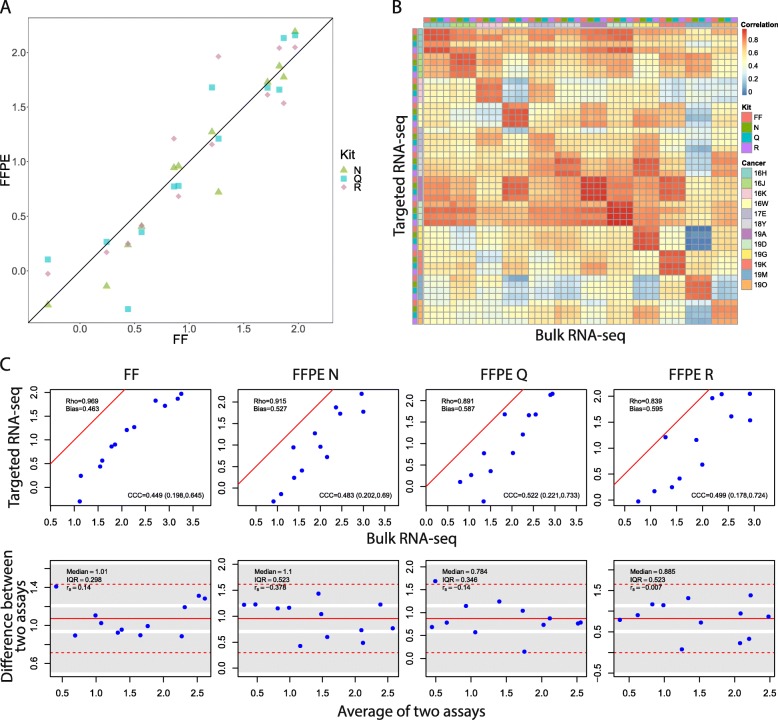
Robustness of targeted sequencing assay for SET_ER/PR_ index. **a** Concordance of SET_ER/PR_ between FFPE and FF samples. **b** Heatmap of correlation matrix between genes in SET_ER/PR_ index calculated on wtRNAseq and targeted RNAseq platforms. **c** Concordance of SET_ER/PR_ signature between two platforms (scatter plots on top and Bland-Altman plots on bottom)

The targeted RNAseq assay from FFPE samples was highly concordant (CCC) with matched FF samples for each extraction method: N (0.96), Q (0.91) and R (0.92) (Fig. 5a). SET_ER/PR_ index measured from targeted sequencing was highly concordant with wtRNAseq for each sample type and extraction method per tumor, more so than between different tumors (Fig. 5b). Different RNA extraction kits for FFPE specimens produced higher correlation of SET_ER/PR_ index (targeted versus wtRNAseq) than different sample types (Fig. 5b). Despite this high correlation, there was linearly biased higher SET_ER/PR_ index from wtRNAseq using all methods (Fig. 5c).

## Discussion

All three FFPE RNA extraction kits require similar hands-on time and yielded similar RNA quantities. However, the purity of extracted RNA varied widely between kits. We observed that when A260/A230 ratio was less than 1, further clean-up by ethanol precipitation was required for downstream customized targeted RNAseq. In this study, there was sufficient RNA purity, not requiring additional clean-up, in 88% (21/24) of FFPE samples extracted with kit N, 75% (15/24) with kit R and 33% (8/24) with kit Q. Although RINs indicated inferior RNA quality from all three FFPE kits, the proportion of RNA molecules of at least 200 bases length was only slightly lower than for FF samples, and the transcript coverage from resultant RNAseq libraries (TIN) was slightly better than FF. Our study design required pooling of libraries from FF and FFPE samples during sequencing, so there was more extensive fragmentation of RNAseq libraries from FF samples than FFPE samples in order to balance the number of reads per sample in each lane of the flow cell, and mitigate technical batch effect on gene expression measurements. That might have contributed to the observed difference in TINs.

All three FFPE RNA extraction kits produced similarly excellent analytical performance compared to FF samples. The cross-linking introduced by fixation may increase the rate of errors during reverse transcription, leading to fewer duplicates and incorrect mapping to intronic regions, as previously observed [12]. Additionally, the non-random fragmentation of FF RNA may cause more duplicates [31]. Intronic reads may also appear due to higher fractions of pre-mRNA with unspliced introns in FFPE [32]. Any observed differences between the FFPE kits were minimal and not statistically significant, whether using the RNA for wtRNAseq or targeted RNAseq assays. The targeted sequencing assay also provided reliable results with the three FFPE RNA extraction kits, and showed only a small (correctable) bias compared to wtRNAseq. We did not expect identical results from these two techniques because they employ very different molecular protocols, and the observed bias illustrates a systematic difference. However, low expressed transcripts were less reliable between technical replicates and less concordant between FFPE and FF samples, and this was not resolved by any of the RNA extraction kits for FFPE samples. These findings are consistent with a general tenet of RNAseq technology: most of noise in the data comes from low read counts [33]. Researchers should consider this issue when selecting genes for molecular assays. Only deeper sequencing of the transcriptome may reveal low abundance transcripts and splice junctions [34], however in many cases it might be too costly unless targeted. Even if targeted, we can still appreciate that pre-analytical conditions might lead to amplification biases unless adequately controlled in the targeted RNAseq procedure.

When applied to wtRNAseq data, the EndoPredict and SET_ER/PR_ index showed excellent analytical performance under different pre-analytical conditions of sample preservation and RNA extraction. Results of recurrence score analysis were less concordant. Notably, 4 of 5 reference genes had lower expression in FFPE samples, i.e. ACTB, GAPDH, GUSB and RPLP0. Others have shown lower expression of GAPDH and ACTB in FFPE samples compared to matched FF samples, using qPCR [16]. In another study, Ct values for GADPH were 2–3 cycles lower for 1-year-old samples than for 10-year-old samples when input RNA amounts were the same [35], suggesting that storage time may affect estimation of GAPDH expression value from FFPE. Our results suggest that customized diagnostic assays must be calibrated to wtRNAseq from matched samples before inferring that RNAseq measurements can be accurately represented.

Among the 18,695 genes analyzed in this study, the results of concordance analysis, differential analysis, replicate analysis and LME analysis identified poorly concordant genes (Additional file 11: Table S5). This poor concordance is mostly driven by higher shift in expression between FF and FFPE samples (median(bias) = 0.79), rather than low correlation (median(r) = 0.86). In concordance analysis we found that genes with high correlation between FF and FFPE tend to have smaller shift in expression (r_s_ = 0.45; *p* < 0.001). The information about shift in expression provided from mixed-effect models analysis (fixed effect estimate), was similar to bias given from concordance analysis (r_s_ = 0.69; *p* < 0.01). Although many genes with different expression level between FF and FFPE were identified, the difference was relatively small (median(|LFC|) = 0.33). We believe that this genome-wide comparison may be highly informative in selecting individual genes for new breast cancer molecular signatures.

Our study was limited to only 12 cancer samples under supervised research collection methods, and does not represent the full diversity of specimen handling and fixation methods in pathology, or among different laboratories extracting RNA or performing RNA sequencing. Also, we could not study pre-analytical effects from prolonged storage of FFPE blocks prior to sectioning – a potentially important factor in retrospective analysis of clinical trial samples. Nevertheless, biospecimen integrity studies (in addition to this) can better inform the selection of reliable transcripts for new breast cancer molecular signatures in at least three scenarios: (i) signature discovery using FF samples with intention to later translate for use with FFPE samples, (ii) use of FF samples to calculate signature discovered on FFPE samples, and (iii) to select genes with consistent expression in FF or FFPE samples.

## Conclusions

The selection of kit to purify RNA from FFPE did not influence the quality of results from wtRNAseq, thus variable reproducibility of gene signatures probably relates to gene selection and possibly algorithm. Targeted RNA sequencing showed promising performance for clinical deployment of quantitative assays in breast cancer FFPE samples, although measurements are not identical to wtRNAseq.

## Supplementary information

**Additional file 1: Table S1.** Storage time and RNA quality of samples used for wtRNAseq and targeted RNAseq.

**Additional file 2: Table S2.** Statistical comparison of RNA quality indices between different sample types and RNA extraction kits.

**Additional file 3: Figure S1.** Comparison of TIN score of individual transcripts between all samples.

**Additional file 4: Table S3.** Statistical comparison of sequencing quality indices between different sample types.

**Additional file 5: Figure S2.** Comparison of coverage along transcript (A) and gene biotype (B) between all samples.

**Additional file 6: Figure S3.** Comparison of results for low (normalized expression < − 7.5) and high (normalized expression > = − 7.5) expression genes from concordance analysis (A) and LME analysis (B and C).

**Additional file 7: Figure S4.** Concordance correlation coefficient (CCC) summarized per chromosome (A) and genomic position within each chromosome (B).

**Additional file 8: Figure S5.** Differential analysis of wtRNAseq data. (A) No. of significant genes (FDR < 0.5) at different log2-fold change level in comparison of FFPE kits and FF samples. (B) Intersection of genes differentially expressed between FFPE kits and FF samples. (C) Intersection of genes differentially expressed between FFPE kits.

**Additional file 9: Table S4.** Summary of concordance and LME analysis for molecular signatures on wtRNAseq data.

**Additional file 10: Figure S6.** Concordance analysis for three molecular signatures. (A) CCC for target genes (red dot) and normalizers (yellow dot) among all analyzed genes (*n* = 18,695). (B) CCC stratified by the role of signature genes (normalizers – red box; target genes – blue box). (C) Concordance of selected signatures (red dot) among distribution of concordance for signatures based on random genes.

**Additional file 11: Table S5.** Results of concordance analysis of gene expression between FFPE and FF samples for all 18,695 genes analyzed in this study.

## Data Availability

The raw wtRNAseq and targeted RNAseq datasets analyzed during the current study are available from the corresponding author on reasonable request.

## Abbreviations

CCC: Concordance correlation coefficient
DV200: Percentage of RNA fragments longer than 200 nucleotides
ERBB2: Human epidermal growth factor 2 receptor
ESR1: Estrogen receptor
FC: Fold change
FF: Fresh frozen
FFPE: Formalin-fixed paraffin-embedded
H&E: Haemotoxylin and eosin
LFC: Log2-fold change
LME: Linear mixed-effects model
mRNA: Messenger RNA
PCA: Principal component analysis
PGR: Progesterone receptor
RIN: RNA integrity number
RNAseq: RNA sequencing
rRNA: Ribosomal RNA
RS: Recurrence score
SET_ER/PR_: Index for sensitivity to endocrine therapy
wtRNAseq: Whole transcriptome RNA sequencing
