# Supplementary figures and images for: The impact of RNA extraction method on accurate RNA sequencing from formalin-fixed paraffin-embedded tissues
Source: BMC Cancer. 2019 Dec 5;19:1189. doi: 10.1186/s12885-019-6363-0 (PMC6896723; doi:10.1186/s12885-019-6363-0)

TIN score

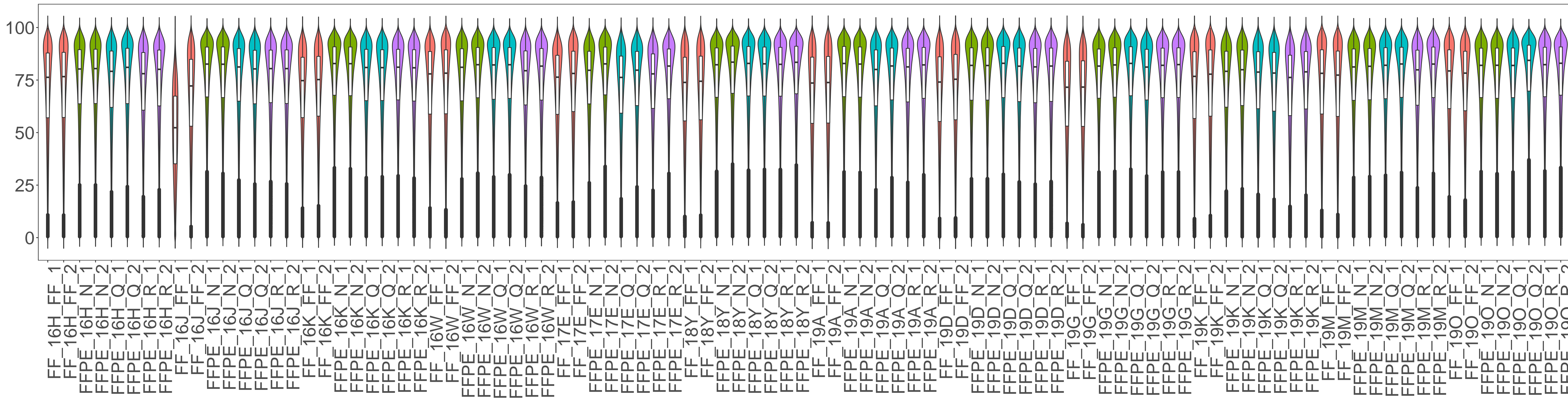

Supplement: Supplementary file 3 — Additional file 3: Figure S1. Comparison of TIN score of individual transcripts between all samples. [file 12885_2019_6363_MOESM3_ESM.pdf]

A

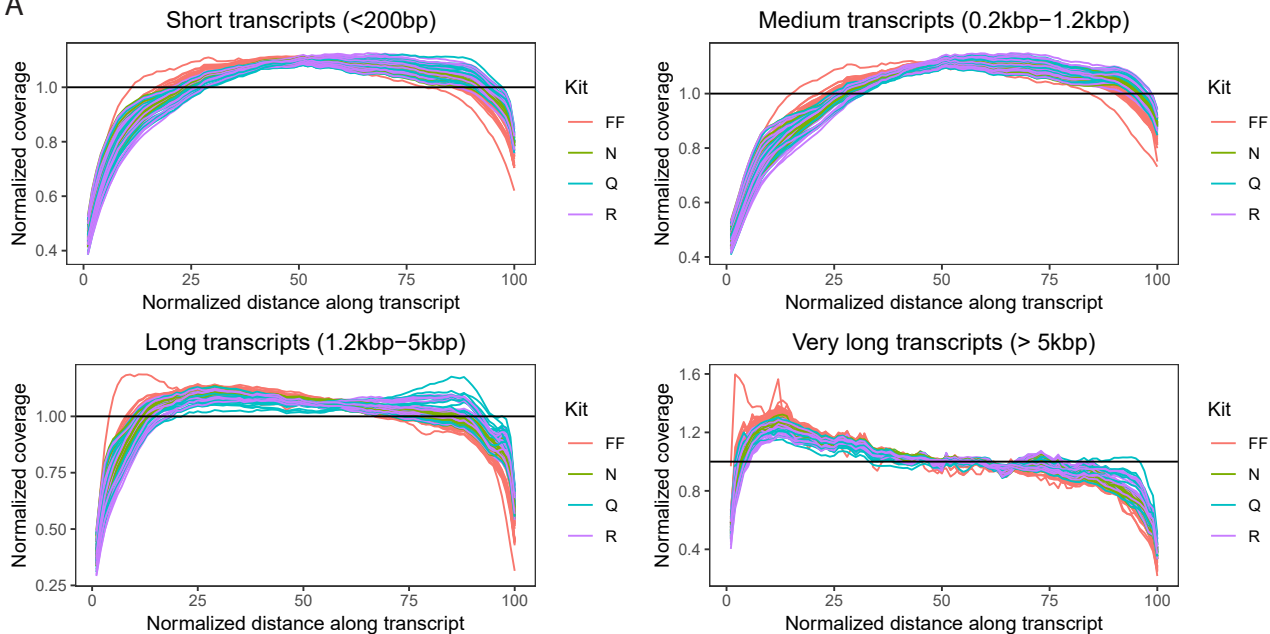

B

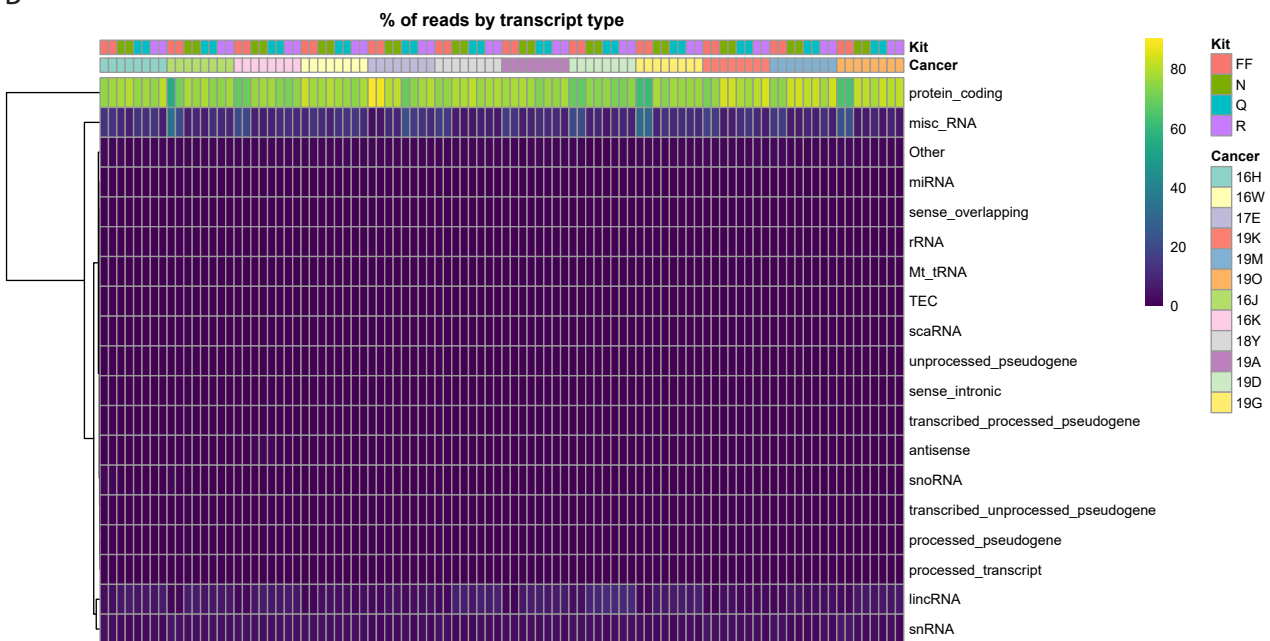

Supplement: Supplementary file 5 — Additional file 5: Figure S2. Comparison of coverage along transcript (A) and gene biotype (B) between all samples. [file 12885_2019_6363_MOESM5_ESM.pdf]

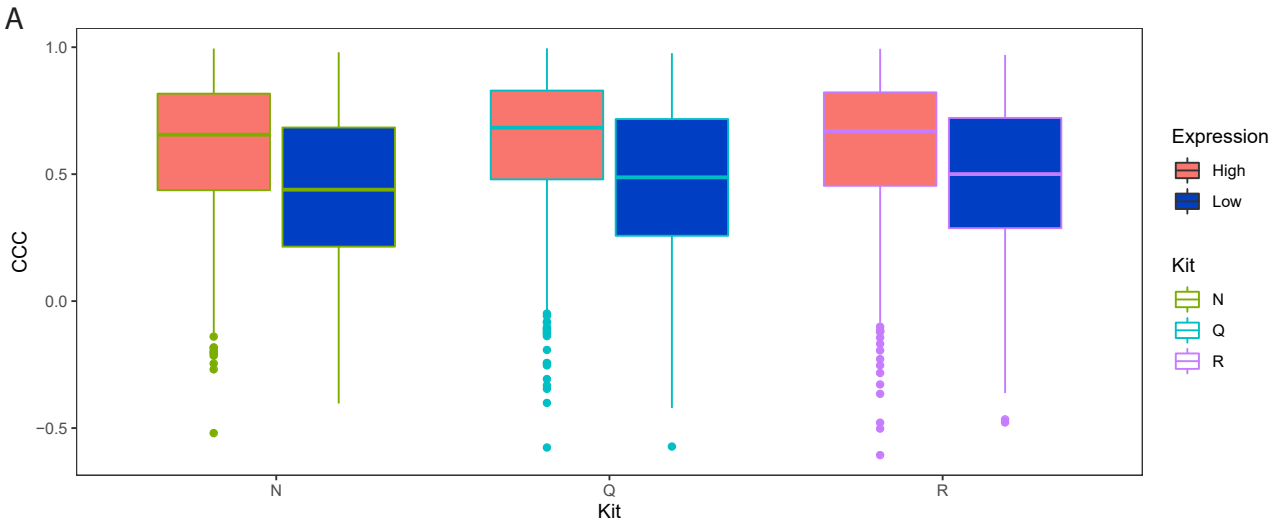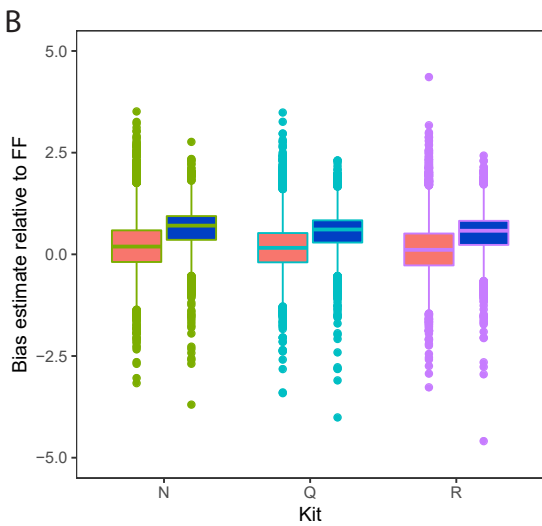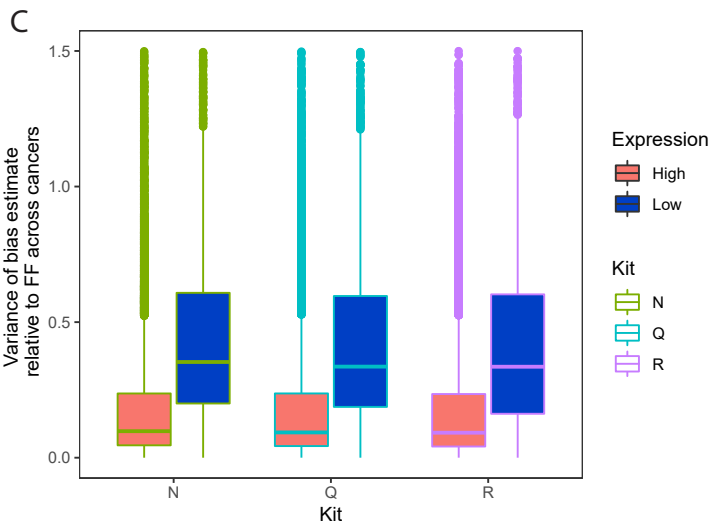

Supplement: Supplementary file 6 — Additional file 6: Figure S3. Comparison of results for low (normalized expression < − 7.5) and high (normalized expression > = − 7.5) expression genes from concordance analysis (A) and LME analysis (B and C). [file 12885_2019_6363_MOESM6_ESM.pdf]

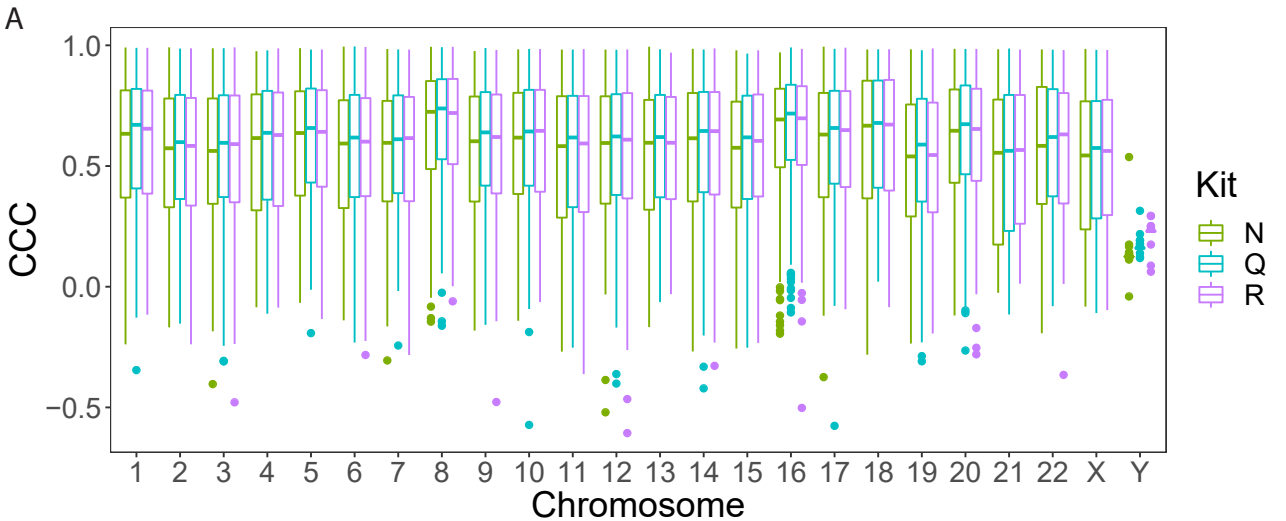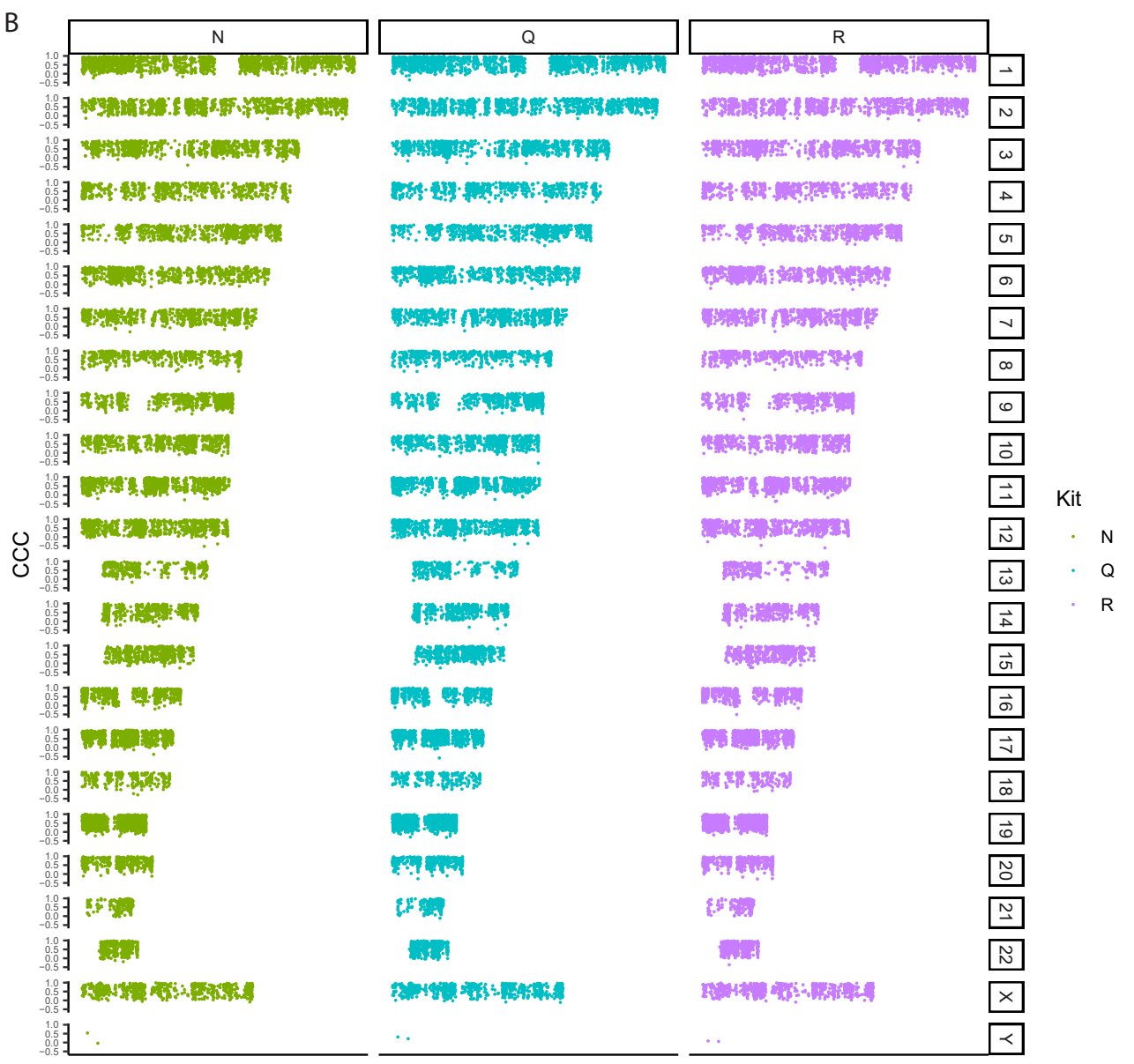

Supplement: Supplementary file 7 — Additional file 7: Figure S4. Concordance correlation coefficient (CCC) summarized per chromosome (A) and genomic position within each chromosome (B). [file 12885_2019_6363_MOESM7_ESM.pdf]

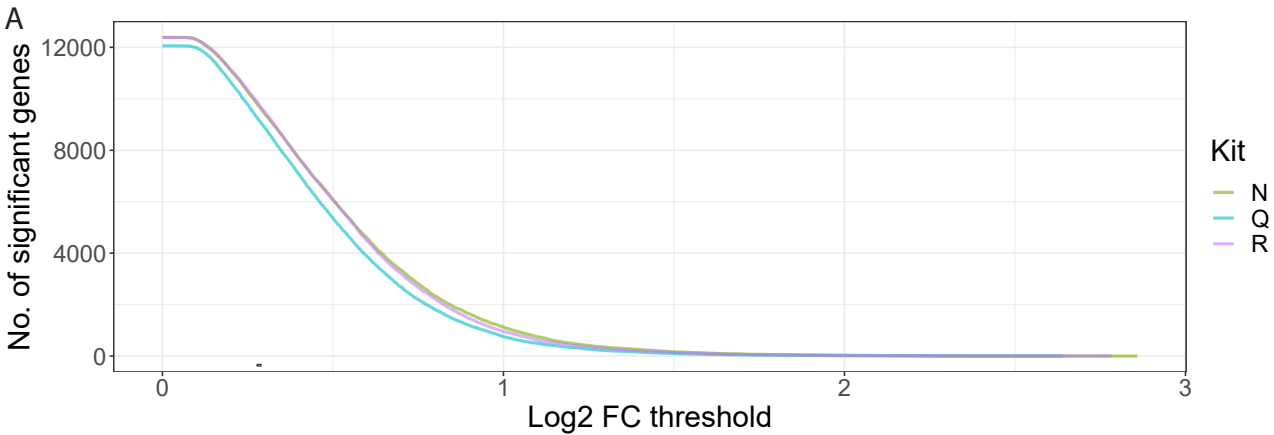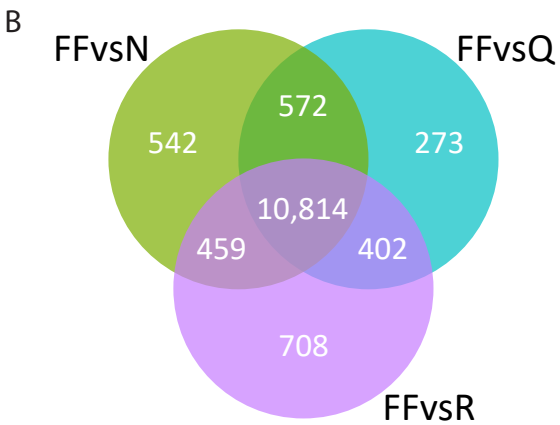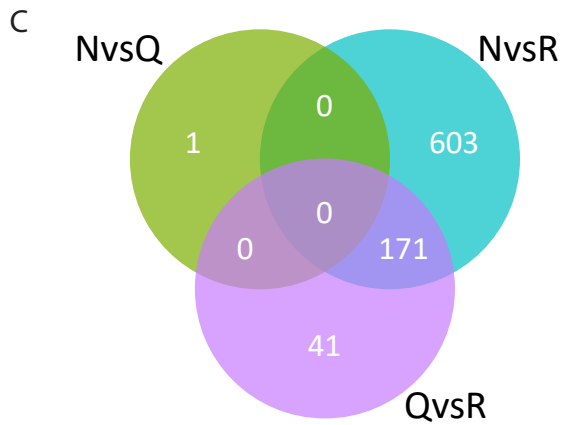

Supplement: Supplementary file 8 — Additional file 8: Figure S5. Differential analysis of wtRNAseq data. (A) No. of significant genes (FDR < 0.5) at different log2-fold change level in comparison of FFPE kits and FF samples. (B) Intersection of genes differentially expressed between FFPE kits and FF samples. (C) Intersection of genes differentially expressed between FFPE kits. [file 12885_2019_6363_MOESM8_ESM.pdf]

A

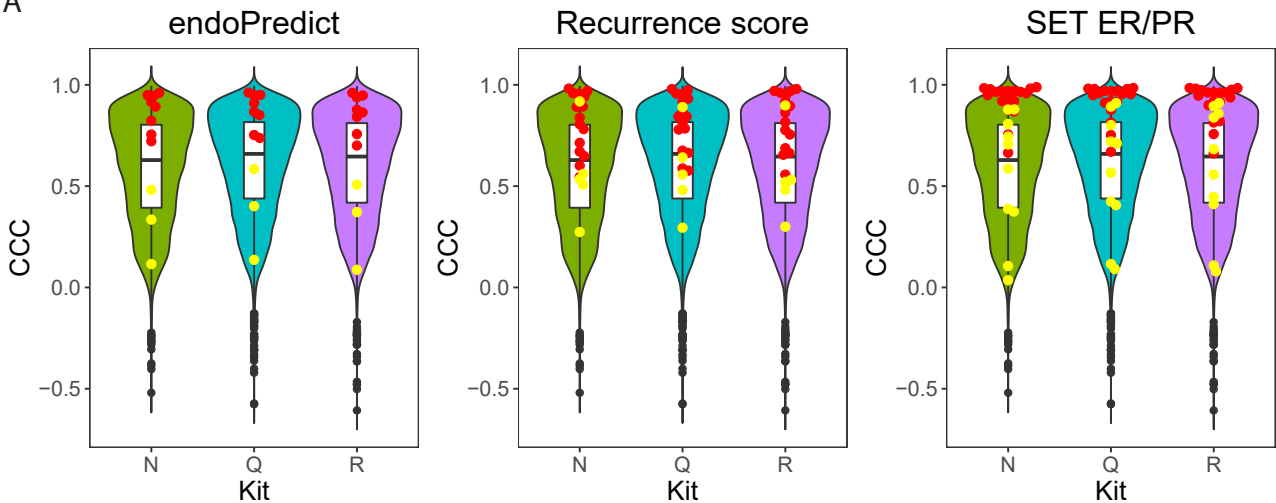

B

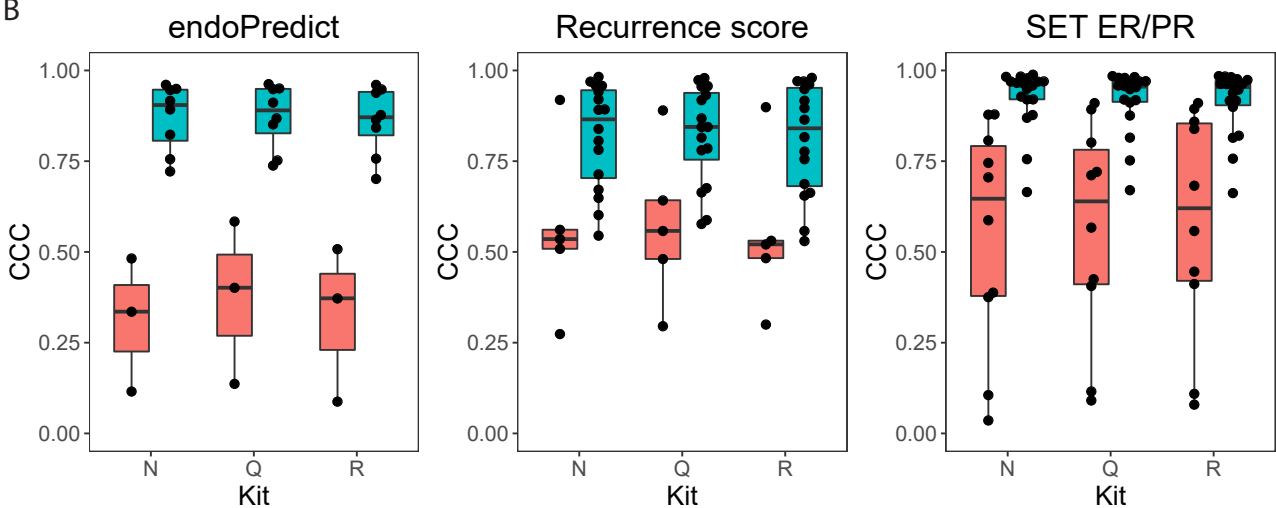

C

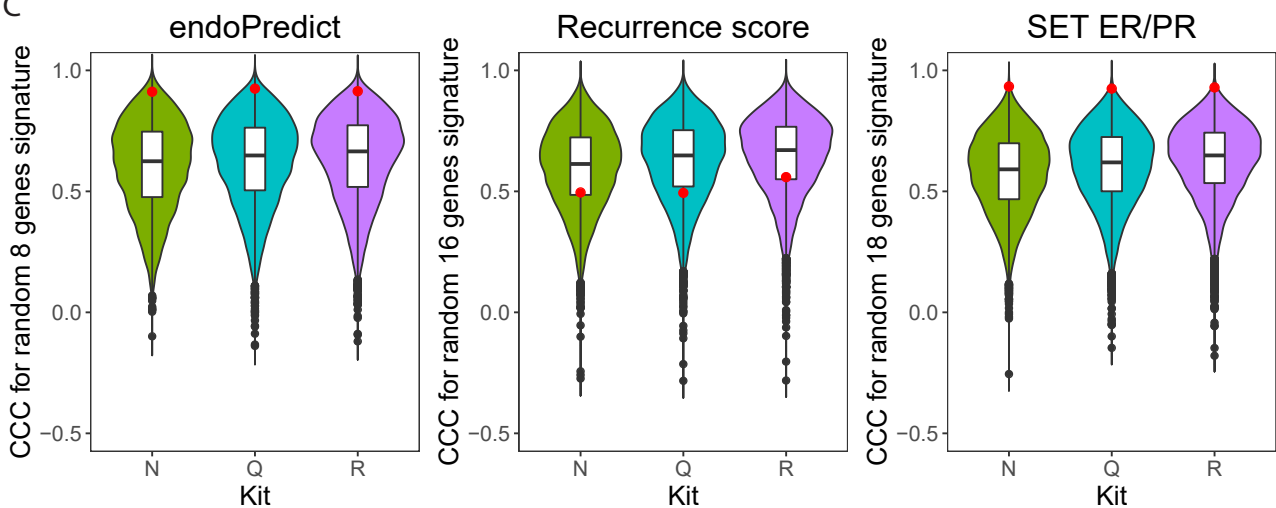

Supplement: Supplementary file 10 — Additional file 10: Figure S6. Concordance analysis for three molecular signatures. (A) CCC for target genes (red dot) and normalizers (yellow dot) among all analyzed genes (n = 18,695). (B) CCC stratified by the role of signature genes (normalizers – red box; target genes – blue box). (C) Concordance of selected signatures (red dot) among distribution of concordance for signatures based on random genes. [file 12885_2019_6363_MOESM10_ESM.pdf]
